# Determination of stanozolol and 3^′^-hydroxystanozolol in rat hair, urine and serum using liquid chromatography tandem mass spectrometry

**DOI:** 10.1186/1752-153X-6-162

**Published:** 2012-12-22

**Authors:** Nawed IK Deshmukh, Gergely Zachar, Andrea Petróczi, Andrea D Székely, James Barker, Declan P Naughton

**Affiliations:** 1 School of Pharmacy and Chemistry, Kingston University, Penrhyn Road, Kingston upon Thames, Surrey, KT1 2EE, UK; 2 Department of Anatomy, Histology and Embryology, Semmelweis University, Budapest IX, Tüzoltó utca 58, H-1450, Hungary; 3 School of Life Sciences, Kingston University, London, UK

**Keywords:** Anabolic androgenic steroid, Doping, Hair analysis, Urinalysis, Serum analysis, LC-MS/MS

## Abstract

**Background:**

Anabolic androgenic steroids, such as stanozolol, are typically misused by athletes during preparation for competition. Out-of-competition testing presents a unique challenge in the current anti-doping detection system owing to logistic reasons. Analysing hair for the presence of a prohibited drug offers a feasible solution for covering the wider window in out-of-competition testing. To assist *in vivo* studies aiming to establish a relationship between drug levels detected in hair, urine and blood, sensitive methods for the determination of stanozolol and its major metabolite 3^′^-hydroxystanozolol were developed in pigmented hair, urine and serum, using brown Norway rats as a model system and liquid chromatography tandem mass spectrometry (LC-MS/MS).

**Results:**

For method development, spiked drug free rat hair, blood and urine samples were used. The newly developed method was then applied to hair, urine and serum samples from five brown Norway rats after treatment (intraperitoneal) with stanozolol for six consecutive days at 5.0 mg/kg/day. The assay for each matrix was linear within the quantification range with determination coefficient (r^2^) values above 0.995. The respective assay was capable of detecting 0.125 pg/mg stanozolol and 0.25 pg/mg 3^′^-hydroxystanozolol with 50 mg hair; 0.063 ng/mL stanozolol and 0.125 ng/mL 3^′^-hydroxystanozolol with 100 μL of urine or serum. The accuracy, precision and extraction recoveries of the assays were satisfactory for the detection of both compounds in all three matrices. The average concentrations of stanozolol and 3^′^-hydroxystanozolol, were as follows: hair = 70.18 ± 22.32 pg/mg and 13.01 ± 3.43 pg/mg; urine = 4.34 ± 6.54 ng/mL and 9.39 ± 7.42 ng/mL; serum = 7.75 ± 3.58 ng/mL and 7.16 ± 1.97 ng/mL, respectively.

**Conclusions:**

The developed methods are sensitive, specific and reproducible for the determination of stanozolol and 3^′^-hydroxystanozolol in rat hair, urine and serum. These methods can be used for *in vivo* studies further investigating stanozolol metabolism, but also could be extended for doping testing. Owing to the complementary nature of these tests, with urine and serum giving information on recent drug use and hair providing retrospective information on habitual use, it is suggested that blood or urine tests could accompany hair analysis and thus avoid false doping results.

## Background

**Figure 1:**
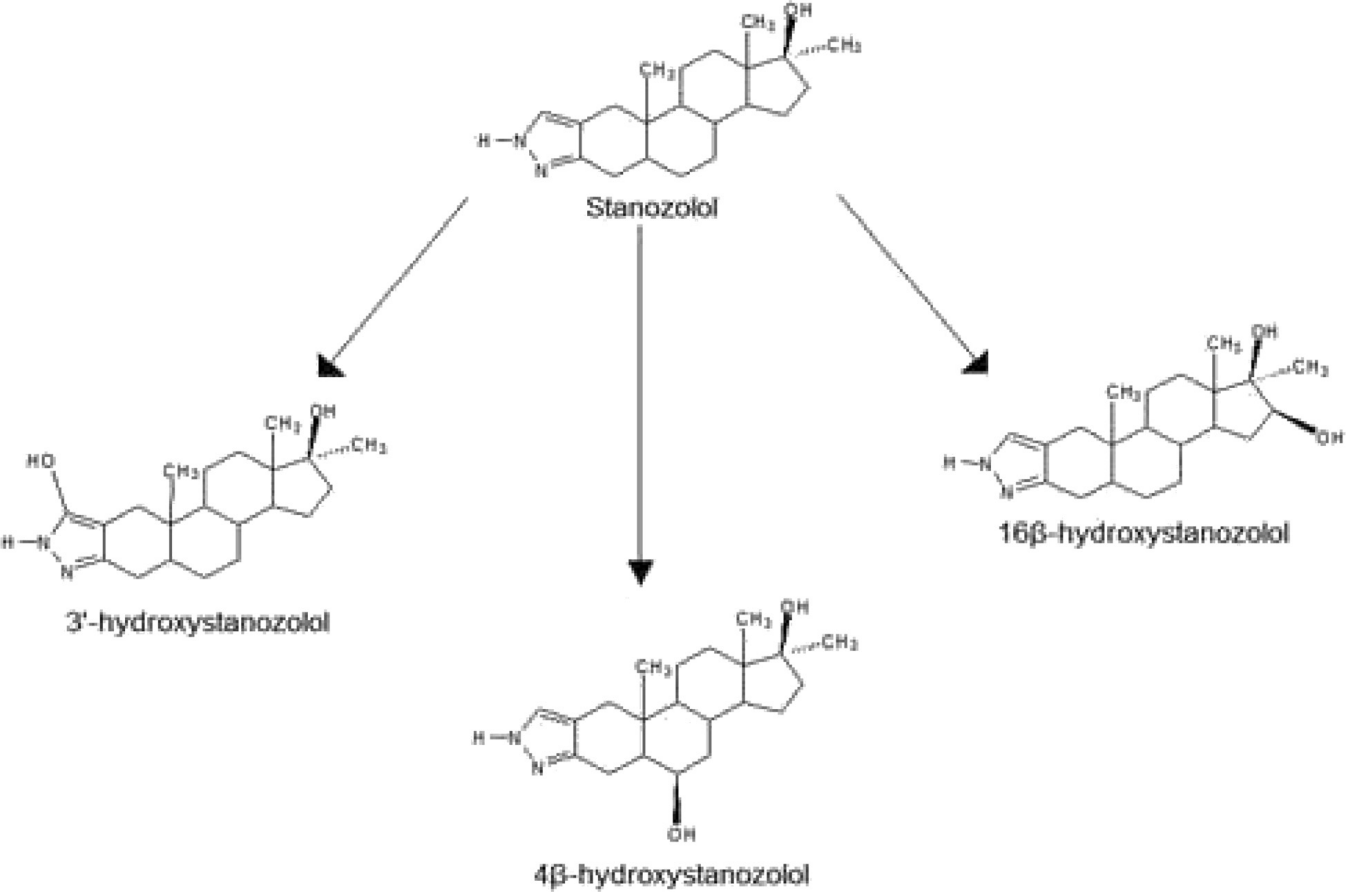
Structures of stanozolol and its major urinary metabolites.

Laboratory statistics of the World Anti-doping Agency (WADA) show that anabolic-androgenic steroids (AAS) account for around 53.6% (average from 2005 to 2010) of all adverse analytical findings in sports [1-6]. Among these, stanozolol is one of the most frequently identified AAS. Stanozolol is a synthetic derivative of the male sex hormone testosterone. According to ‘The 2013 Prohibition List’ of the WADA code, stanozolol belongs to class S1.1a and its use is prohibited both in- and out-of-competition [7]. Doping with stanozolol is suspected if the urinary concentration of stanozolol and/or its metabolites exceeds 2 ng/mL [8]. Three of the major metabolites of stanozolol are reported to be 3^′^-hydroxystanozolol, 4β-hydroxystanozolol and 16β-hydroxystanozolol (Figure 1), which are excreted in urine mainly as glucuronide conjugates [9]. Amongst these, the urinary level of 3^′^-hydroxystanozolol, post deglucuronidation, is routinely used for screening stanozolol misuse [8-10].

Since stanozolol and 3^′^-hydroxystanozolol are structurally different from most AAS, they can be more difficult to detect in urine than other AAS [11], and thus require bespoke methods. Depending on the dose administered, once in the body, stanozolol gets rapidly metabolised and the metabolites are generally detected in urine until *ca.* 6 days [11]. Thus, urinalysis generally fails to determine the long term history of an individual’s drug use [12], which is a major hindrance in cases of performance-enhancing drugs used in preparation for competition. Stanozolol, along with other AAS, is a so called ‘training drug’ which is taken for a prolonged period, typically in cycles, during preparation, in order to obtain the desired performance-enhancing effects [13,14]. Furthermore, urinalysis also fails to distinguish between chronic use and single, accidental exposure of drugs [15].

The major elimination and deactivation pathway of AAS and their phase I metabolites is through glucuronide conjugation (phase II metabolism), mainly catalysed by the enzyme UGT2B17, followed by excretion in urine [16-19]. However, inter-individual and inter-ethnic variations in the prevalence of deletion polymorphism in the gene coding of the UGT2B17 enzyme have been reported, which eventually influence the urinary excretion of AAS and potentially lead to false-negative doping results [20,21]. It has also been reported that the glucuronidation activity of UGT2B17 and other UGTs towards AAS is inhibited by commonly used anti-inflammatory drugs like diclofenac and ibuprofen, *in vitro*[22-26]. Common dietary substances such as red wine [27], white tea and green tea [28] have also shown similar inhibitory effects in *in vitro* studies. Although the inhibitory effect is yet to be examined and reported *in vivo*, these *in vitro* results indicate that concomitant use of such over-the-counter medication or common dietary products with AAS may lead to impaired urinary excretion of AAS and their metabolites.

Considering that such genetic and metabolic variations may limit the efficacy of urinalysis in testing doping, it can be suggested that urinalysis, if used as a stand-alone test, is susceptible to confounding doping results [11-13,16-21]. Owing to the growing number of doping cases with AAS [1-6], there is an ever-increasing need to develop new methods to detect drug doping. The current anti-doping regime can be reinforced by employing additional biological samples like blood and hair analysed in tandem with urine. Since impaired glucuronidation leads to reduction in the urinary excretion rate of AAS, it can be assumed that the levels of unconjugated AAS and their phase I metabolites in the systemic circulation will be elevated and thus higher levels of AAS and their phase I metabolites will be available to get incorporated into hair and other body tissues [21]. Hair analysis has been used in the past for detecting drug use [29-32] as it predominantly favours the direct detection of parent AAS and determines a retrospective history of drug use. Thus, hair analysis and blood analysis [33] can provide complementary information to urinalysis to prevent false doping results.

However, to investigate this option further, *in vivo* studies are required to establish a relationship between the drug levels detected in hair, urine and blood. To the best of our knowledge, such studies for the determination of stanozolol and its major metabolite, 3^′^-hydroxystanozolol in the three matrices together are, as yet, not reported in the literature. Thus, the aim of this work was to take a step forward by developing liquid-chromatography tandem mass spectrometry (LC-MS/MS) based methods which are capable of determining the concentrations of stanozolol and 3^′^-hydroxystanozolol in pigmented hair, urine and blood serum samples of stanozolol-treated rats.

In the past, *in vivo* studies have been reported where administration of a single high dose of stanozolol (60 mg/kg) to guinea pigs afforded the detection of stanozolol in hair [34,35], whereas it was not possible to detect 3^′^-hydroxystanozolol [34]. Since, metabolites are generally difficult to detect in hair, it is reasonable to assume that a single-dose treatment may not be sufficient to investigate whether levels of metabolites can be determined in hair. However, multiple doses of stanozolol along with sensitive analytical methods can provide this key information. Thus, as a preliminary step, a 6 day treatment period was used in this study to improve the potential for detecting the metabolites in hair. Athletes typically administer AAS at doses ranging from 3 mg/kg to 25 mg/kg to increase muscle mass, which are 10 to 100 fold higher than the therapeutic doses [36]. Thus, in line with previous steroid-abuse rat studies [36-39], the present study was designed with a daily dose of 5.0 mg/kg for 6 consecutive days, followed by analysing hair, urine and sera samples using newly developed LC-MS/MS methods.

### Experimental

#### Chemicals, reagents and consumables

Reference standards for stanozolol, 3^′^-hydroxystanozolol, 3^′^-hydroxystanozolol glucuronide, 3^′^-hydroxystanozolol D3 and stanozolol D3 were purchased from LGC standards (Teddington, UK). Sodium hydrogen phosphate heptahydrate, sodium phosphate monobasic dihydrate, sodium hydroxide, formic acid, hydrochloric acid, LC-MS grade water, acetonitrile, methanol, HPLC grade dichloromethane, pentane, chloroform and ethylacetate were purchased from Sigma Aldrich (Poole, UK). β-glucuronidase from *E. Coli* (Cat. No. 03707598001, Lot No. 12438921) was purchased from Roche Diagnostics (Burgess Hill, UK). All chemicals were of analytical-reagent grade and were used without further purification. For the animal experiment, stanozolol, ketamine (2.5%) and xylazine (Rompun, 2%) were purchased from Desma (Madrid, Spain), Kőbányai Gyógyszerárugyár (Budapest, Hungary) and Haver-Lockhart laboratories (Kansas, US) respectively. A SB C-18 column (2.1 mm, 50 mm, 1.8 μm) and 0.2 μm inline filter was purchased from Agilent (Stockport, UK). Syringe driven 0.2 μm PTFE filters were purchased from Millipore (Watford, UK). Silanised glass inserts were purchased from Capital Analytical (Leeds, UK). Silanised, amber, glass vials were purchased from Sigma Aldrich (Poole, UK).

### Animals

Male, brown Norway rats were purchased from Charles River laboratories (Sulzfeld, Germany). Each animal weighed around 280–340 g and was approximately 5 months old. All animals were kept in an animal house located in Semmelweis University, Budapest, Hungary. Animals were housed in groups of three individuals in standard laboratory cages. Rats were kept in a constant room temperature environment with an alternating 12-h light–dark cycle. Food and water were available *ad-libitum*.

### Administration of stanozolol and sample collection

Five rats kept in standard lab cages under 12/12 light/dark cycle were administered with stanozolol (in saline) intra-peritoneally [34], at a dose of 5.0 mg/kg/day for six consecutive days. The dose of stanozolol selected was in line with previous steroid studies using rat models [36-39] and considered equivalent to levels abused by humans on a milligram per kilogram of body weight basis [36,37]. Hair, urine and blood samples were collected on the 7^th^ day of the study, *i.e.* one day after stopping the stanozolol treatment.

The growth rate of rat hair was tested prior to the treatment regime by shaving the back of the experimental animals and the sampling protocol was adjusted accordingly.

Urine was collected by gently pressing the abdomen. Blood was taken from the tail vein. Blood samples were left to clot for 45 to 60 minutes and then centrifuged (at 1000 × g for 10 minutes at room temperature) to harvest serum. Before collecting blood and urine samples, the animals were anaesthetised with a mixture of ketamine and xylazine. Two weeks before the experiment, the entire dorsal surface of the animal was shaved to the skin with an electric shaver and drug-free control hair was collected and preserved. Exactly the same dorsal surface was sampled on the 7^th^ day of the experiment to avoid any diluting effect of the hair grown before the stanazolol treatment period. Drug-free blood and urine samples were also collected before the experiment was initiated. Serum and urine samples obtained were stored at −80°C. Hair samples were stored in sealed, clean envelopes at room temperature. The administration of stanozolol and sample collection were conducted under the institutional license of Department of Anatomy, Histology and Embryology, Semmelweis University, Budapest, Hungary in accordance with the EC Council directives on laboratory animals (86/609/EEC). Samples were analysed in Kingston University.

### Sample preparation

#### Hair samples

Hair samples were initially decontaminated by rinsing twice with 2 mL dichloromethane for two minutes at room temperature. After decontamination, hair samples were allowed to air dry and then pulverised using a ball mill. Fifty milligrams of decontaminated hair powder was incubated with 1 mL 1 M sodium hydroxide at 95°C for 10–15 minutes in the presence of deuterated internal standards (ISs) stanozolol D3 and 3^′^-hydroxystanozolol D3. After cooling, the homogenate was neutralised with 1 M hydrochloric acid, followed by addition of 2 mL of 0.2 M phosphate buffer (pH 7.0).

#### Serum and urine samples

Serum and urine samples were thawed and vortex mixed. A 100 μL aliquot of each was used for analysis.

#### Enzymatic hydrolysis of glucuronide conjugates

The enzyme β-glucuronidase was used for the enzymatic hydrolysis of glucuronide conjugates to determine the total concentration (glucuronide conjugated + unconjugated) of stanozolol and 3^′^-hydroxystanozolol in each matrix (hair, urine and serum). For this step, and, in a similar manner to the hair samples, the serum and urine samples were also neutralised by mixing with 1 mL of 0.2 M phosphate buffer (pH 7.0). The neutralised solutions of hair, serum and urine were hydrolysed by incubation with 50 μL of β-glucuronidase at 50°C for two hours in the presence of internal standards [9]. After cooling, the samples were purified by performing liquid–liquid extraction (LLE).

#### Sample purification

LLE was carried out by using a mixture of pentane, chloroform and ethylacetate (4 mL in total) in the ratio 3:2:1 v/v/v. The mixture was vortex mixed for 20 seconds and then centrifuged at 4000 × g for 20 minutes at 4°C. The organic layer was transferred into a clean, silanised, glass vial and evaporated at 40°C using a gentle stream of nitrogen gas. The dried residue was reconstituted with 100 μL methanol. The reconstituted solution was filtered through a 0.2 micron PTFE membrane filter, prior to injecting (3 μL) into the LC-MS/MS system.

### Liquid chromatographic-tandem mass spectrometry

The analysis of stanozolol and its metabolite 3^′^-hydroxystanozolol was carried out using an LC-MS/MS system, which comprised of a 1260 infinity LC system (Agilent, Wokingham, UK) coupled to a 6430 triple quadrupole mass spectrometer (Agilent, Wokingham,UK). The LC system comprised of a binary pump, automatic degasser, column heater and 1290 infinity thermostated autosampler. The analytical column used was a SB C-18 column (2.1 mm, 50 mm, 1.8 μm), kept in a column oven at 45°C. A 0.2 micron inline filter was installed prior to the column to prevent the analytical column from blocking. Mobile phase solvents comprised of water with 0.001% v/v formic acid as solvent A and 50:50 mixture of acetonitrile and methanol as solvent B. The flow rate of mobile phase through the column was 300 μL/min. The gradient flow composition is shown in Table 1.

**Table 1:** **Chromatograms of stanozolol and 3**^**′**^**-hydroxystanozolol extracted from (a) hair, (b) urine and (c) serum at LLOQ concentration levels**

**LC run time (minutes)**	**Solvent A Water (0.001% formic acid)**	**Solvent B Acetonitrile: methanol (50:50)**
0	60	40
1	60	40
2	15	85
5	0	100
6	0	100
7	60	40
15	60	40

The mass spectrometer was equipped with an electrospray ionisation (ESI) source, which was operated in positive ion mode. The protonated molecules, [M + H]^+^, of stanozolol (*m*/*z* 329.5), 3^′^-hydroxystanozolol (*m/z* 345.5), stanozolol D3 (*m/z* 332.5) and 3^′^-hydroxystanozolol D3 (*m/z* 348.5) were used as precursor ions for collision induced dissociation (CID) for MS-MS analysis. The mass spectrometer was operated in multiple reaction monitoring (MRM) mode to monitor the precursor ions and the diagnostic product ions of each analyte and IS. The MRM transitions, collision energies and retention times of each analyte and internal standard are detailed in Table 2.

**Table 2:** **Retention times, MRM transitions and collision energies of stanozolol, 3**^′^**-hydroxystanozolol, stanozolol D3 and 3**^′^**-hydroxystanozolol D3**

**Compounds**	**Retention time (min)**	**MRM transitions**	**Collision energy (eV)**
Stanozolol	6.0	329.5 > 81.1	50
		329.5 > 121.1	46
3^′^-Hydroxystanozolol	5.6	345.5 > 97.1	50
		345.5 > 121.1	42
Stanozolol D3	6.1	332.2 > 81.2	50
3^′^-Hydroxystanozolol D3	5.8	348.5 > 97.1	50

For the optimum ionisation of analytes, the following mass spectrometric conditions were applied: capillary voltage, 4000 V; drying gas temperature, 325°C; drying gas flow rate, 10 L/min; nebulising gas pressure, 35 psi and fragmentor voltage of 125 V. The mass spectrometric parameters were optimised using the Masshunter optimizer software (version B.03.01). The LC-MS/MS system was controlled by the Masshunter workstation software (LC/MS data acquisition, version B.03.01).

### Method validation

The validation of the analytical methods was performed according to the Food and Drug Administration (FDA) guidelines [40], by determining accuracy, precision, lower limits of quantification (LLOQ), lower limits of detection (LLOD), linearity, selectivity, and extraction recoveries [41,42]. Drug-free rat hair, urine and serum samples were used for method development and validation. Samples for calibration curves were prepared by spiking known amounts of stanozolol, 3^′^-hydroxystanozolol and ISs (stanozolol D3 and 3^′^-hydroxystanozolol D3) to drug-free hair, urine and serum. Quality control (QC) samples were prepared similarly at three concentration levels (for each matrix) distributed over the linear range. Calibration curves were prepared for each matrix by plotting the analyte to IS ratio against the known concentrations of analyte in each sample. The analyte to IS ratio for each analyte was obtained by dividing the peak area of analyte by the peak area of the IS. Samples for calibration curves and quality controls were treated in a way similar to unknowns. The linearity of the method was investigated by using linear regression analysis.

The accuracy of each assay was determined by analysing QC samples at three concentration levels in replicates (N = 6, per concentration level) and comparing the mean calculated values with the respective nominal concentration values. Intra-day precision was determined by measuring 6 replicates per concentration level, on the same day. Inter-day precision was assessed by analysing 6 replicates per concentration level, on three consecutive days. Intra-day and inter-day precision of the method was characterised in terms of relative standard deviation (RSD, %). The limits of acceptable variability were set at 15% for all the concentrations, except at LLOQ, for which 20% was accepted. LLOD was defined as the lowest concentration of the analyte which gave a peak response equivalent to three times the background noise [i.e. signal to noise ratio (S/N) ≥ 3]. LLOQ was defined as the lowest amount of analyte which gave a peak response with a S/N ≥ 10 and which could be measured with adequate precision and accuracy (RSD less than 20% and an inaccuracy ±20%) [40].

The selectivity of the method was determined by analysing the drug-free samples of hair, urine and serum in replicates and confirming the absence of any detectable peaks at the retention times of stanozolol, 3^′^-hydroxystanozolol and ISs. The extraction recovery for each analyte was determined at three concentration levels by replicate analysis (N = 6) of blank matrices (urine, serum and hair) spiked with known concentrations of analytes and ISs and then extracted as described above. The analyte to internal standard peak area ratios obtained after extraction were then compared with analyte to internal standard peak area ratios of standard solutions prepared in methanol at the same final concentrations. To determine matrix effects, blank hair, urine and serum samples from different animals were extracted as described above. In order to consider only the matrix effect and not losses during the extraction procedure, the blank extracts were spiked with known concentrations of analytes and ISs after the extraction step, followed by analysis. The resulting peak areas of stanozolol, 3^′^-hydroxystanozolol and ISs were then compared with the peak areas of standard solutions of stanozolol, 3^′^-hydroxystanozolol and ISs at the same theoretical concentrations.

## Results and discussion

### Method development

**Figure 2:**
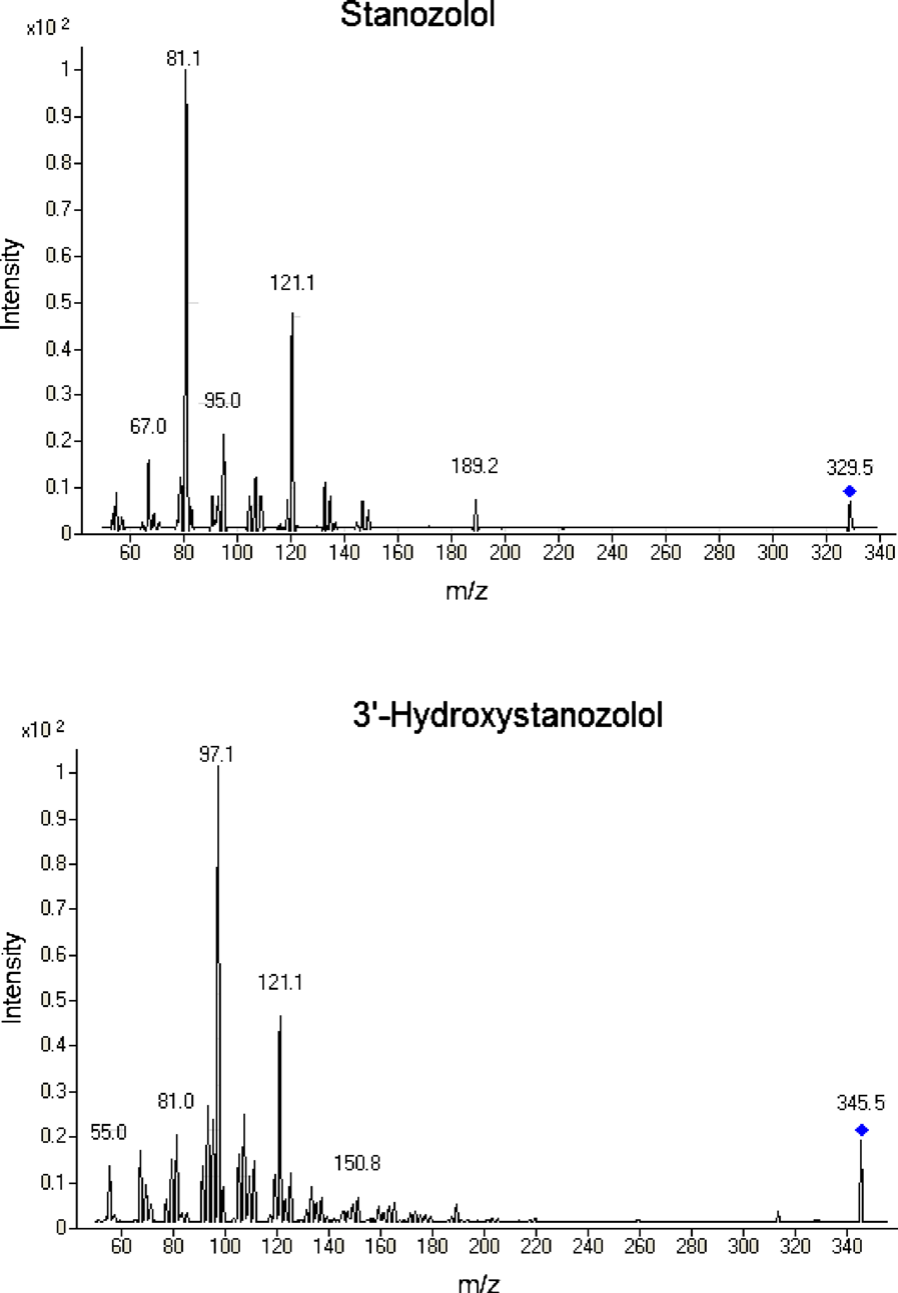
**Product ion mass spectra (full scan) of stanozolol and 3**^**′**^**-hydroxystanozolol.**

Both stanozolol and 3^′^-hydroxystanozolol were detected and quantified on the basis of their retention time and MRM transitions (Table 2). The most abundant product ions that were monitored for stanozolol were m/z 81.1 and 121.1, whereas for 3^′^-hydroxystanozolol, the most abundant product ions that were monitored were m/z 97.1 and 121.1. Figure 2 represents the product ions mass spectra (full scan) of stanozolol and 3^′^-hydroxystanozolol. Operating the mass spectrometer in MRM mode enhanced the method selectivity, sensitivity and specificity. Stanozolol D3 and 3^′^-hydroxystanozolol D3 were used as internal standards for stanozolol and 3^′^-hydroxystanozolol respectively. Internal standards were used to compensate for any: i) ionisation suppression, ii) variations in the instrument response from injection to injection and iii) loss of analytes during sample preparation.

Use of different mobile phase solvents was investigated. For instance, use of water as solvent A in combination with methanol or acetonitrile or a mixture of methanol and acetontrile (50:50) as solvent B was examined. Different gradient and isocratic mobile phase compositions were investigated. Addition of formic acid (0.001% v/v, 0.01% v/v and 0.1% v/v) to solvent A and/or solvent B was also investigated. Optimum sensitivity and excellent peak shapes for all analytes and ISs were obtained when water with formic acid (0.001% v/v) was used as solvent A and a mixture of acetonitrile and methanol (50:50) was used as solvent B under the gradient conditions shown in Table 1. It was observed that when formic acid was added to solvent A and/or solvent B at concentrations ≥ 0.01% v/v, there was a drastic reduction in the sensitivity of all analytes and ISs (up to 50%). However, when formic acid was added only to water (solvent A) at a concentration of 0.001% v/v, there was no effect on the sensitivity and peak shapes.

For hair analysis, alkali digestion was employed for the extraction of drugs from hair matrix. Alkali digestion ensures complete dissolution of the hair matrix and hence it is generally known to give good recoveries of drugs entrapped in the hair matrix. However, a potential drawback of complete dissolution of hair is that the components of hair matrix in solution may interfere with the analysis. Thus, to reduce the unwanted matrices that may affect the analysis, sample purification was carried out using LLE. The extraction efficiencies of different solvents like pentane, hexane, chloroform, ethyl acetate and ethanol, and their combinations were investigated. It was found that a mixture of pentane, chloroform and ethyl acetate in the ratio 3:2:1 v/v/v facilitated maximum recovery of analytes and ISs. Also, owing to the hair decontamination step employed (using dichloromethane), no external interferences were observed. The LLE step employed was also efficient for the extraction of stanozolol, 3^′^-hydroxystanozolol and ISs from the urine and serum samples. Under the analytical conditions employed, there were no matrix interferences that affected the analysis of stanozolol and 3^′^-hydroxystanozolol in hair, urine and serum. The enzymatic hydrolysis of glucuronide conjugates of stanozolol and 3^′^-hydroxystanozolol was carried out. This ensured that the total concentration (glucuronide conjugated plus unconjugated) of stanozolol and 3^′^-hydroxystanozolol could be determined in each matrix [9]. The conditions employed for enzymatic hydrolysis step (incubation temperature, time and pH) were optimised using 3^′^-hydroxystanozolol glucuronide. Complete hydrolysis of the glucuronide conjugate was achieved when the pH of the sample solution was adjusted to 7, followed by incubation with β-glucuronidase (50 μL) at 50°C for 2 hours.

### Method validation

**Figure 3:**
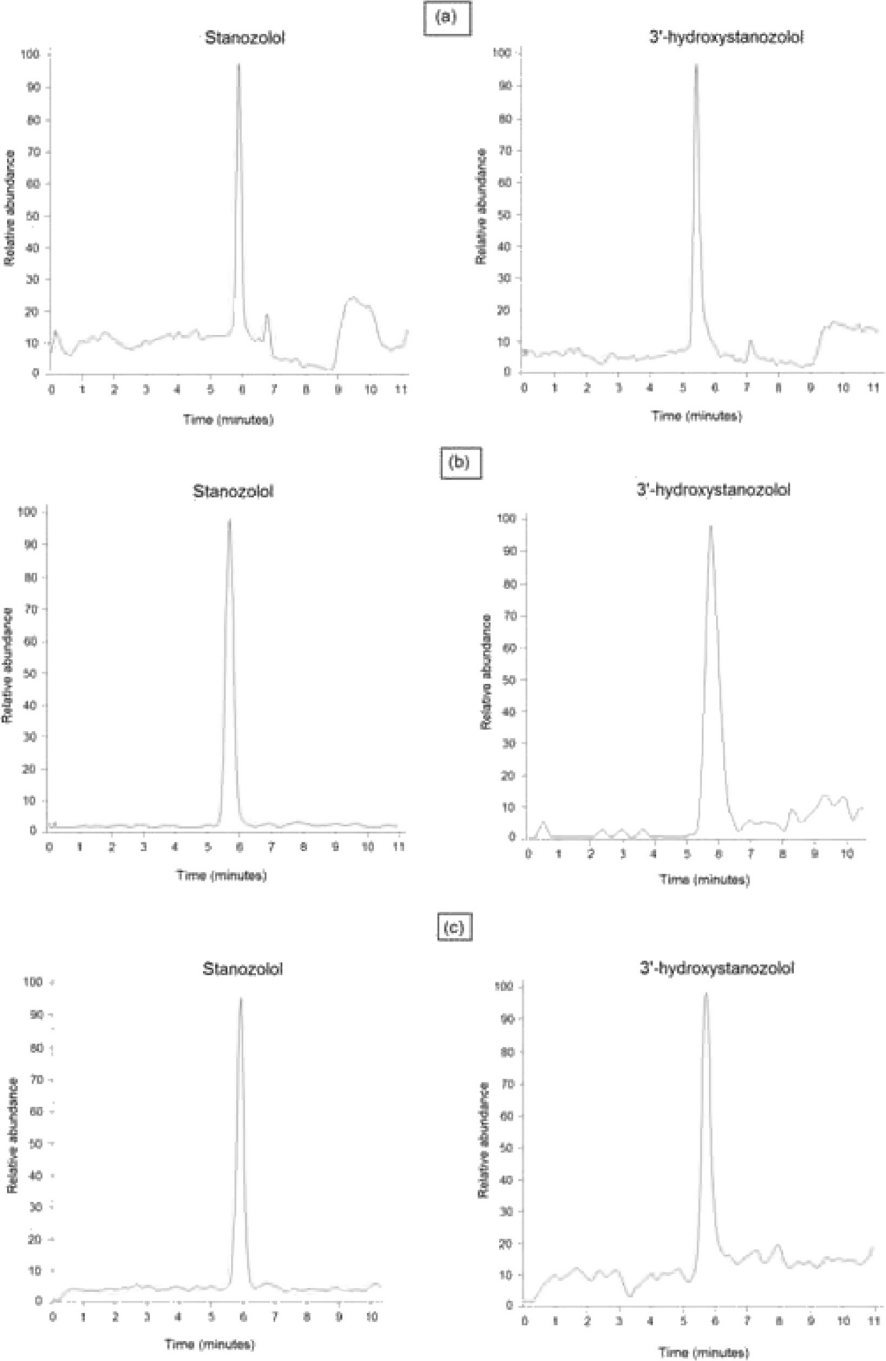
**Chromatograms of stanozolol and 3**^**′**^**-hydroxystanozolol extracted from (a) hair, (b) urine and (c) serum at LLOQ concentration levels.**

The validation results are within the limits set by the FDA guidelines [40]. The methods were selective and specific for unambiguous determination of stanozolol and 3^′^-hydroxystanozolol in all three matrices. Suppression or enhancement of analyte ionisation owing to co-eluting components of matrices was not observed. Excellent peak shape was achieved for stanozolol, 3^′^-hydroxystanozolol, stanozolol D3 and 3^′^-hydroxystanozolol D3. Figure 3 represents chromatograms of stanozolol and 3^′^-hydroxystanozolol extracted from hair, urine and serum at LLOQ concentration levels. Typical calibration curves of stanozolol and 3^′^-hydroxystanozolol in all three matrices are provided in Additional file 1.

#### Hair

The assay for hair analysis was linear in the range 0.5 - 400 pg/mg for both stanozolol and 3^′^-hydroxystanozolol. The determination coefficient (r^2^) values were found to be higher than 0.9986 for all calibration curves. The method was capable of detecting (LLOD) stanozolol and 3^′^-hydroxystanozolol in hair at concentrations as low as 0.125 pg/mg and 0.25 pg/mg respectively when *ca.* 50 mg hair was processed. The LLOQ level of both stanozolol and 3^′^-hydroxystanozolol was found to be 0.5 pg/mg. The accuracy, intra-day precision and inter-day precision results of the assay are detailed in Table 3. The extraction recoveries of both compounds (at three concentration levels) from hair are presented in Table 4.

**Table 3:** **Accuracy, intra-day precision and inter-day precision of the assay for detecting stanozolol and 3**^**′**^**-hydroxystanozolol in rat hair**

**Compounds**	**Concentration (pg/mg)**	**Level**	**Precision RSD (%)**		**Accuracy (%)**
			**Intra-day**	**Inter-day**	
Stanozolol	2.5	Low	3.4	2.8	105.0
	20	Medium	1.5	1.3	103.4
	100	High	1.2	2.8	100.7
3^′^-Hydroxystanozolol	2.5	Low	1.5	4.1	97.3
	20	Medium	3.6	3.4	101.9
	100	High	3.8	5.3	100.5

**Table 4:** **Extraction recovery results of stanozolol and 3**^**′**^**-hydroxystanozolol from hair**

**Compounds**	**Concentration (pg/mg)**	**% Extraction recovery (N = 6)**
Stanozolol	0.5 (LLOQ)	100.84
	2.5	103.53
	10	105.39
3-Hydroxystanozolol	0.5 (LLOQ)	88.51
	2.5	107.99
	10	102.64

#### Urine

The assay for urinalysis was linear in the range 0.125 - 25 ng/mL for stanozolol and 0.25 - 25 ng/mL for 3^′^-hydroxystanozolol. The determination coefficient (r^2^) values were found to be higher than 0.9959 for all runs. The method was capable of detecting (LLOD) stanozolol and 3^′^-hydroxystanozolol at concentrations as low as 0.063 ng/mL and 0.125 ng/mL urine respectively, when only 100 μL aliquot of urine was processed. The LLOQ levels of stanozolol and 3^′^-hydroxystanozolol were found to be 0.125 ng/mL and 0.25 ng/mL urine respectively. Table 5 summarises the accuracy, intra-day precision and inter-day precision results of the assay. The extraction recoveries from urine at three concentration levels are presented in Table 6.

**Table 5:** **Accuracy, intra-day precision and inter-day precision of the assay for detecting stanozolol and 3**^**′**^**-hydroxystanozolol in rat urine**

**Compounds**	**Concentration (ng/mL)**	**Level**	**Precision RSD (%)**		**Accuracy (%)**
			**Intra-day**	**Inter-day**	
Stanozolol	0.5	Low	10.4	6.3	90.6
	2.5	Medium	4.6	7.0	105.6
	5	High	4.1	4.0	111.4
3^′^-Hydroxystanozolol	0.5	Low	4.9	6.2	89.7
	2.5	Medium	7.1	5.0	109.2
	5	High	6.4	5.1	104.2

**Table 6:** **Extraction recovery results of stanozolol and 3**^**′**^**-hydroxystanozolol from urine**

**Compounds**	**Concentration (ng/mL)**	**% Extraction recovery (N = 6)**
Stanozolol	0.125 (LLOQ)	107.87
	2.5	111.77
	10	109.64
3-Hydroxystanozolol	0.25 (LLOQ)	91.44
	2.5	107.92
	10	111.82

#### Serum

The serum assay showed good linearity within the quantification range 0.25 - 100 ng/mL for both stanozolol and 3^′^-hydroxystanozolol, with determination coefficient (r^2^) values higher than 0.9981. The method was capable of detecting (LLOD) stanozolol and 3^′^-hydroxystanozolol in serum at concentrations as low as 0.063 ng/mL and 0.125 ng/mL respectively when only 100 μL aliquot of serum was processed. The LLOQ level of both stanozolol and 3^′^-hydroxystanozolol was found to be 0.25 ng/mL. The accuracy, intra-day precision and inter-day precision results of the assay are detailed in Table 7. The extraction recoveries are presented in Table 8.

**Table 7:** **Accuracy, intra-day precision and inter-day precision of the assay for detecting stanozolol and **3^**′**^**-hydroxystanozolol in rat serum**

**Compounds**	**Concentration (ng/mL)**	**Level**	**Precision RSD (%)**		**Accuracy (%)**
			**Intra-day**	**Inter-day**	
Stanozolol	1.25	Low	4.4	3.0	111.0
	10	Medium	1.4	1.6	108.4
	50	High	1.7	1.4	90.4
3^′^-Hydroxystanozolol	1.25	Low	4.3	6.4	98.0
	10	Medium	3.5	3.9	102.3
	50	High	4.8	5.3	91.9

**Table 8:** **Extraction recovery results of stanozolol and **3^**′**^**-hydroxystanozolol from serum**

**Compounds**	**Concentration (ng/mL)**	**% Extraction recovery (N = 6)**
Stanozolol	0.25 (LLOQ)	113.24
	2.5	101.92
	10	109.42
3-Hydroxystanozolol	0.25 (LLOQ)	95.36
	2.5	111.03
	10	105.29

### Application of the method to real samples

The developed methods were employed for determining the total (glucuronide-conjugated plus un-conjugated) concentration of stanozolol and its metabolite 3^′^-hydroxystanozolol in rat hair, urine and serum samples. Table 9 represents the average concentrations (three replicates) of stanozolol and 3^′^-hydroxystanozolol in hair, urine and serum samples of each rat. The average concentrations of stanozolol and 3^′^-hydroxystanozolol in rat hair were found to be 70.18 ± 22.32 pg/mg and 13.01 ± 3.43 pg/mg respectively. The average ratio of concentrations of stanozolol to 3^′^-hydroxystanozolol in hair was found to be 5.38 ± 0.93. Variations observed in the concentration of stanozolol and 3^′^-hydroxystanozolol amongst individual animals could be owing to differences in their metabolic pattern. Furthermore, difference in the amount of water consumed by animals can also lead to variations in the levels of drugs in their body. The results indicate that stanozolol gets preferentially incorporated in hair relative to its metabolite 3^′^-hydroxystanozolol. These findings are in agreement with previous reports [32]. In the past, researchers have found it difficult to detect 3^′^-hydroxystanozolol in hair. Cirimile *et al.* reported the detection of stanozolol in scalp hair of a bodybuilder who declared to be a regular user of stanozolol [43]. However, 3^′^-hydroxystanozolol was not detectable in hair under their analytical conditions. Similarly, in another study carried out by Shen *et al.*, stanozolol was detectable in guinea pig hair after administering stanozolol at a single high dose of 60 mg/kg, whereas, 3^′^-hydroxystanozolol was not detectable [34]. However, the method presented here was capable of detecting stanozolol and 3^′^-hydroxystanozolol in rat hair after administering stanozolol for 6 days at a dose of 5.0 mg/kg/day that is considered equivalent to those levels abused by athletes [36-39]. Thieme *et al.* have reported a case where both stanozolol and 3^′^-hydroxystanozolol were detectable in the hair of a bodybuilder using gas chromatography high resolution mass spectrometry (GC-HRMS), after sample derivatisation [44]. In the past GC-MS and GC-HRMS have been frequently employed for the detection of AAS [43-46]. The major disadvantage of such technique is that it requires a laborious and expensive sample derivatisation step. Generally, the derivatives are unstable and susceptible to thermal decomposition during analysis, thus affecting the reproducibility of the method. In contrast, LC-MS/MS normally does not require any additional derivatisation step. Thus, LC-MS/MS can be considered as a more economical and feasible approach for analysing AAS [29,30,47].

**Table 9:** **Concentrations of stanozolol and 3**^**′**^**-hydroxystanozolol in rat hair, urine and serum samples**

**Matrix**	**Animal**	**Stanozolol**	**3**^**′**^**-Hydroxystanozolol**	**Ratio**
Hair (pg/mg)				
	1	46.57 ± 0.17	10.96 ± 0.15	4.25
	2	47.25 ± 1.07	9.13 ± 0.12	5.18
	3	82.58 ± 2.67	16.92 ± 0.37	4.88
	4	77.98 ± 1.03	11.72 ± 0.30	6.65
	5	96.54 ± 1.02	16.29 ± 0.07	5.93
	Mean	70.18 ± 22.32	13.01 ± 3.43	5.38 ± 0.93
Urine (ng/mL)				
	1	5.20 ± 0.09	13.54 ± 0.43	0.38
	2	0.20 ± 0.01	0.36 ± 0.02	0.56
	3	0.17 ± 0.02	5.07 ± 0.01	0.03
	4	0.70 ± 0.01	8.51 ± 2.41	0.08
	5	15.41 ± 0.08	19.49 ± 0.22	0.79
	Mean	4.34 ± 6.54	9.39 ± 7.42	0.37 ± 0.32
Serum (ng/mL)				
	1	5.91 ± 0.00	7.25 ± 0.29	0.82
	2	4.69 ± 0.01	7.62 ± 0.27	0.62
	3	13.01 ± 0.01	8.76 ± 0.67	1.49
	4	5.24 ± 0.06	3.81 ± 0.27	1.38
	5	9.90 ± 0.03	8.38 ± 0.22	1.18
	Mean	7.75 ± 3.58	7.16 ± 1.97	1.09 ± 0.37

In urine, the average concentrations of stanozolol and 3^′^-hydroxystanozolol were found to be 4.34 ± 6.54 ng/mL and 9.39 ± 7.42 ng/mL respectively. The average urinary ratio of stanozolol concentration to 3^′^-hydroxystanozolol concentration was found to be 0.37 ± 0.32. The results indicate that the urinary concentrations of stanozolol are comparatively lower than 3^′^-hydroxystanozolol, as expected. However, in serum the average ratio of concentrations of stanozolol to 3^′^-hydroxystanozolol was found to be 1.09 ± 0.37. Thus, suggesting that both compounds can be detected in serum at similar concentration levels and with equal ease.

The results suggest that the newly developed LC-MS/MS based methods are capable of detecting and quantifying total concentration (glucuronide conjugated plus unconjugated) of stanozolol and its major metabolite, 3^′^-hydroxystanozolol in hair, urine and serum samples of brown Norway rats after administering stanozolol for 6 days at a dose (5.0 mg/kg/day), and this is in line with steroid studies using rat models [36-39]. Future studies may expand the stanozolol treatment period to 3 or more weeks to mimic typical athlete use, along with experimenting with different stanozolol doses and conditions. These newly developed methods can assist *in vivo* studies designed to further investigate the metabolism of stanozolol. Urinalysis can provide information on whether UGT substrates/inhibitors and deletion polymorphism in the UGT2B17 gene reduce the glucuronidation rate (phase II metabolism) of stanozolol and 3^′^-hydroxystanozolol, as impaired glucuronidation has been reported to reduce the urinary concentrations of AAS. It can be assumed that, owing to compromised urinary excretion, the serum levels of unconjugated stanozolol and 3^′^-hydroxystanozolol can get elevated [21]. Thus, potentially greater amounts of stanozolol and 3^′^-hydroxystanozolol will be available to get incorporated in hair. Hence, these methods can assist in investigating the potential application of hair analysis and serum analysis to provide complementary information when the urinary excretion of stanozolol and 3^′^-hydroxystanoozlol is impaired.

## Conclusions

To our knowledge, the detection of stanozolol and 3^′^-hydroxystanozolol in rat hair, urine and serum at such low concentration levels using LC-MS/MS, has been reported here for the first time. Using the newly developed methods presented here, future research can carry out *in vivo* studies to further investigate stanozolol metabolism, thus making an important step towards understanding of the array of factors that may confound urinalysis results. Also, these methods can be extended by analysing human hair, urine and serum samples in tandem to provide a pattern of drug use and this can be useful for testing doping with stanozolol and other commonly abused AAS. Hair can provide retrospective information on an individual’s drug use and this can be used in out-of-competition testing. However, information of current drug-use, if important, can be obtained by urine and blood serum analyses. Thus, when the three tests are used in combination, useful information on an individual’s drug use can be obtained and false doping results can be prevented.

## Abbreviations

AAS: Anabolic androgenic steroids; CID: Collision induced dissociation; FDA: Food and Drug Administration; GC-HRMS: Gas chromatography high resolution mass spectrometry; GC-MS: Gas chromatography mass spectrometry; ISs: Internal standards; LC-MS/MS: Liquid chromatography tandem mass spectrometry; LLE: Liquid-liquid extraction; LLOQ: Lower limit of quantification; LLOD: Lower limit of detection; MRM: Multiple reaction monitoring; QC: Quality control; RSD: Relative standard deviation; UGT2B17: Uridine diphosphate-glucuronosyltransferase 2B17; WADA: World Anti-Doping Agency.

## Competing interests

The authors declare that they have no competing interests.

## Authors’ contribution

AP, JB and DPN initiated the study. GZ, NIKD and AP designed the study. GZ and ADSz conducted the animal experiments. The method development and sample analyses were conducted by NIKD who prepared the draft paper. All authors contributed to data analyses and to finalising the manuscript. All authors have read and approved the final version.

## Supplementary Material

Additional file 1 **Figure S1.** Calibration curves of stanozolol and 3^′^-hydroxystanozolol in (**a**) hair, (**b**) urine and (**c**) serum. Click here for file
