# Supplementary figures and images for: Determination of stanozolol and 3′-hydroxystanozolol in rat hair, urine and serum using liquid chromatography tandem mass spectrometry
Source: Chem Cent J. 2012 Dec 22;6:162. doi: 10.1186/1752-153X-6-162 (PMC3542005; doi:10.1186/1752-153X-6-162)

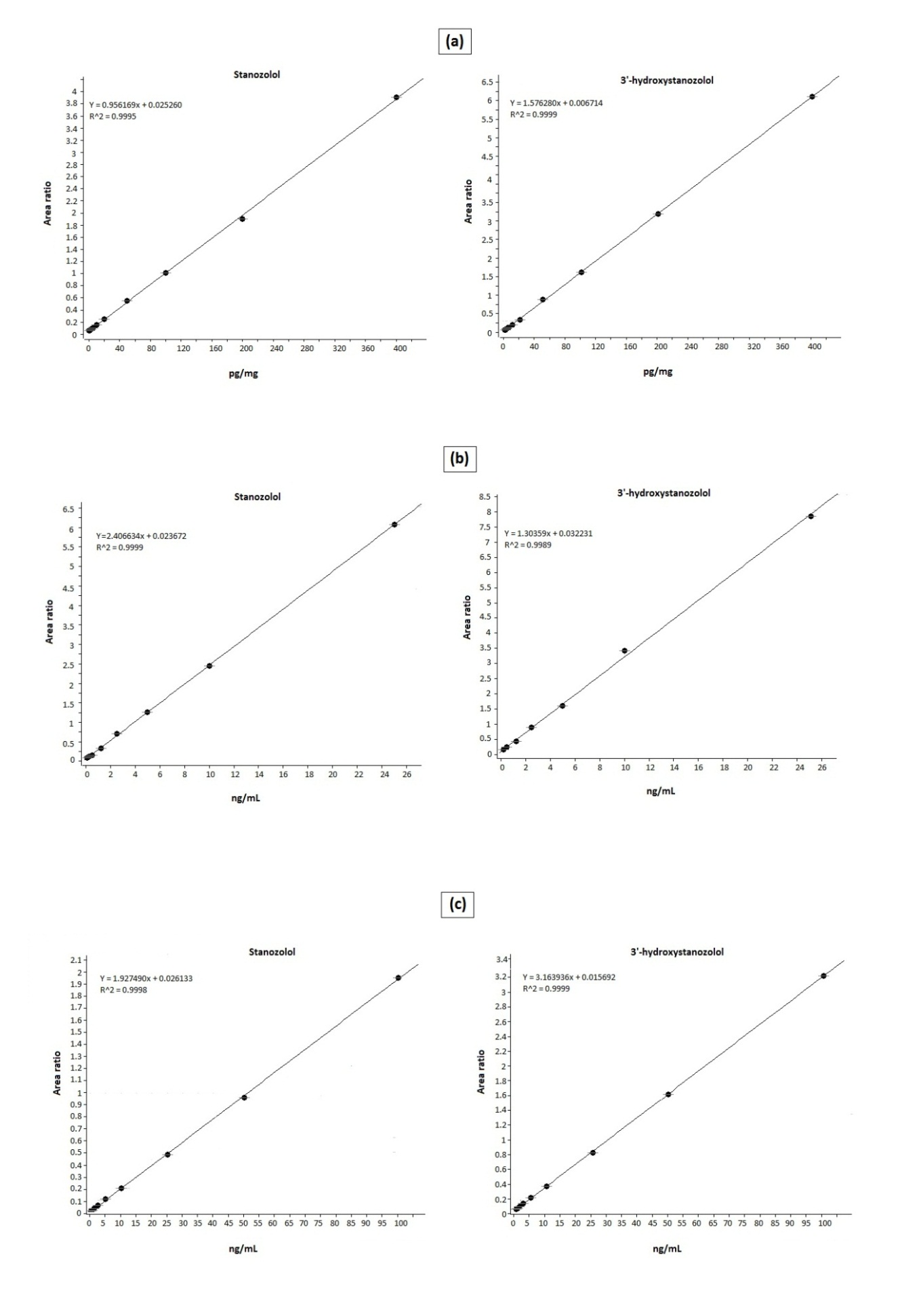


Figure S1: Calibration curves of stanozolol and 3'-hydroxystanozolol in (a) hair, (b) urine and (c) serum

Supplement: Additional file 1 — Figure S1. Calibration curves of stanozolol and 3′-hydroxystanozolol in (a) hair, (b) urine and (c) serum. [file 1752-153X-6-162-S1.docx]
